# Personal Social Networks of Community-Dwelling Oldest Old During the Covid-19 Pandemic—A Qualitative Study

**DOI:** 10.3389/fpubh.2021.770965

**Published:** 2021-12-24

**Authors:** Jenni Kulmala, Elisa Tiilikainen, Inna Lisko, Tiia Ngandu, Miia Kivipelto, Alina Solomon

**Affiliations:** ^1^Faculty of Social Sciences (Health Sciences) and Gerontology Research Center (GEREC), Tampere University, Tampere, Finland; ^2^Division of Clinical Geriatrics, Department of Neurobiology, Care Sciences and Society, Center for Alzheimer Research, Karolinska Institutet, Stockholm, Sweden; ^3^Population Health Unit, Finnish Institute for Health and Welfare, Helsinki, Finland; ^4^Department of Social Sciences, University of Eastern Finland, Kuopio, Finland; ^5^Faculty of Sport and Health Sciences and Gerontology Research Center (GEREC), University of Jyväskylä, Jyväskylä, Finland; ^6^Institute of Clinical Medicine, School of Medicine, University of Eastern Finland, Kuopio, Finland; ^7^Ageing Epidemiology Research Unit, School of Public Health, Imperial College London, London, United Kingdom; ^8^Clinical Trials Unit, Theme Aging, Karolinska University Hospital, Stockholm, Sweden; ^9^Institute of Public Health and Clinical Nutrition, University of Eastern Finland, Helsinki, Finland; ^10^Neurocenter, Department of Neurology, Kuopio University Hospital, Kuopio, Finland

**Keywords:** COVID-19, social connectedness, social relationship, older people, oldest old, personal networks, qualitative study

## Abstract

The COVID-19 pandemic and its related restrictions have affected the everyday life of older people. Advanced age is a significant predisposing factor for a more severe COVID-19 infection, increasing the risk for hospitalization and mortality. Even though restrictions have been, thus, well-grounded, they may also have had detrimental effects on the social well-being of older people. Personal networks and social activity are known protective factors against the premature decline in health and functioning, and it is widely acknowledged that social isolation increases feelings of loneliness, poor quality of life, and even the risk for diseases and disabilities among older adults. This qualitative study investigated changes in personal networks among community-dwelling oldest-old individuals (persons aged 80 and over) during the first and second waves of the COVID-19 pandemic in Finland. The data is part of the Cardiovascular Risk Factors, Aging, and Dementia (CAIDE85+) study, which is an ongoing large longitudinal population-based study in Finland. In this qualitative sub-study, we analyzed fifteen in-depth telephone interviews using directed content analyses and identified five types of changes in personal social networks during the pandemic. In type 1, all social contacts were significantly reduced due to official recommendations and fear of the virus. Type 2 included modified ways of being socially active i.e., by deploying new technology, and in type 3, social contacts increased during the lockdown. In type 4, personal social networks were changed unexpectedly or dramatically due to a death of a spouse, for example. In type 5, we observed stable social networks, which had not been affected by the pandemic. At an individual level, one person could have had different types of changes during the pandemic. These results highlight the heterogeneity of the oldest olds' personal social networks and changes related to them during the exceptional times of the COVID-19 pandemic. Social activity and personal networks play an important role in the well-being of the oldest old, but individual situations, needs, and preferences toward personal social networks should be taken into account when planning social activities, policies, and interventions.

## Introduction

For many older people, the year 2020 challenged the ability to maintain personal social networks. During the COVID-19 outbreak in Finland in March 2020, strict recommendations were put in action, and people aged 70 and above were considered as one of the significant risk groups for severe COVID-19 infection. Therefore, they were asked to stay at home and avoid all face-to-face social contacts. Finland managed to protect this high-risk group, and the total number of deaths due to the virus remained relatively low. Therefore, due to safety reasons, restrictions in social contacts were well-grounded. Even though the restrictions most likely saved lives, the effects of these restrictions on personal networks and to other aspects of the well-being of older adults are still not widely known. This qualitative study aimed to gain a deeper understanding of what kinds of changes in personal social networks the community-dwelling oldest-old persons (persons aged 80 and older) experienced during the first and second waves of the global COVID-19 pandemic in Finland. This study sheds light on the personal social networks of the oldest old persons and their lived experiences during the pandemic.

The ability to gain and maintain social contacts and personal networks plays an important role in both health and well-being across the life course. Living a socially active life—meeting family and friends, attending group activities, and participating actively in society—is linked with several health benefits, including the decreased risk of chronic conditions (1, 2) and higher mental well-being (3), as well as better perceived quality of life and positive feelings and emotions (4). Social activities may reduce the need for health care (5) and are even associated with a lower risk of premature death (6). Among older adults, belonging to a meaningful group after retirement can maintain physical activity and consequently bring greater long-term physical health benefits (7). Also, among people who are considered as being at increased risk of frailty, being socially active seems to be protective from health deterioration and also the decline of subjective well-being (8). In this study, we examine social activity from the perspective of personal social networks, which we understand as nodes of diverse social relations embedded in the everyday lives of older people. Several factors in personal social networks, including the lack of social connections, non-married status, living alone, and low quality of social relationships, on the other hand, increase the risk of loneliness (9), which is common in older age. It has been found that even up to 40% of Finnish older adults aged 80 and above may experience loneliness at least sometimes (10). Loneliness is associated with poorer physical and mental health (11, 12) increased need for care (13) and early mortality (14). Studies from many countries have shown that during the COVID-19 pandemic, loneliness among older adults increased, which indicates that the pandemic has had negative effects on older people's social relations and social activity (15). A study among Swiss older adults (aged 65 and older) by Macdonald and Hülür (16) showed that older adults with larger social networks, a higher number of social contacts, and the possibility for social support experienced less loneliness during the pandemic emphasizing the supportive and protective effect of social relationships. Especially, being satisfied with communication during the COVID-19 pandemic seemed to protect from feelings of loneliness (16).

Among the oldest old, commonly referring to persons aged 80 and above, changes in personal social networks during the global pandemic have not been widely studied. Since social networks and contacts tend to decrease with increasing age also in normal situations due to losses of spouse and friends, everyday life in the shadow of the COVID-19 pandemic has likely had significant effects on the personal networks of the oldest old. Few studies reporting these changes in social activity during the pandemic have been published. For example, a review by Lebrasseur et al. (17) synthesized the existing research on the impact of the pandemic and associated isolation on older adults. This review showed that the pandemic resulted in decreased social life and fewer in-person social interactions and these changes had a negative effect on the quality of life and depression rates increased. Decreased social activity was also associated with difficulties in accessing services, sleep disturbances, and a reduction of physical activity (17).

So far, only a few studies have used qualitative methods to investigate personal networks, changes in social activities, and reactions to restrictions from the perspective of older adults. A qualitative interview study by Goethals et al. (18) investigated the decline in physical activity among older adults during the pandemic from the perspective of professionals who provide or lead physical activity. From their perspective, a significant decrease in attendance in physical activity groups was observed among older adults (18). In another qualitative study conducted in the Netherlands (19), independently living older adults reported that they started to use more digital technologies to maintain social contacts. Part of them suffered from a lack of social contacts, while some participants had no problems with the restrictions. These relatively vital community-dwelling participants were able to adapt to a decrease in social contacts and did not report a significant impact on their socio-emotional well-being. Further, Whitehead and Torossian (20) identified 20 stress categories and 21 joy/comfort categories in their qualitative study assessing older adults' experiences of the COVID-19 pandemic. Isolation and related loneliness were among the commonly reported stressors, but on the contrary, the most reported sources of joy and comfort were contacts with family and friends and also digital social contacts and hobbies (20). Our study also showed that both face-to-face and remote social contacts with family, friends, and acquaintances were an important factor in contributing to everyday life meaningfulness during the pandemic (21). Social contacts are one of the key determinants in terms of well-being also during the exceptional times of this global pandemic. This study aimed to gain a more in-depth understanding of how life in the shadow of a global pandemic affected personal social networks among the oldest old community-dwelling people.

## Materials and Methods

The data for this study is part of the Cardiovascular Risk Factors, Aging, and Dementia (CAIDE) study including older adults who live in the Eastern part of Finland. Participants come originally from five population-based cohorts who were assessed in their midlife in 1972–1992 (22). Altogether, 2,000 individuals were invited for follow-up studies in 1998 and 2005–2008, and an additional 445 individuals in 2005–2008. In 2020–2021, all eligible individuals (still alive and living in the study area; *n* = 806) were invited for the CAIDE85+ study. Invitations to take part in the CAIDE COVID-19 sub-study were sent in July 2020 for all individuals who had participated in the CAIDE85+ main assessment and given their consent for further research contact (*n* = 189) and individuals who had not yet participated in the CAIDE85+ main assessment (*n* = 90). Altogether, 103 (37%) community-dwelling CAIDE85+ participants provided answers to the COVID-19 survey. From this group, 15 persons were invited to participate in qualitative telephone interviews. The interviews were conducted from August 20, 2020 onwards, with the last interview being conducted on the December 1, 2020. The interviewed participants represented a variety of life situations and health. The current study reports data from these qualitative interviews. Each participant or their legally acceptable representative signed the informed consent to participate in the study. The ethical approvement for the CAIDE study has been granted by The Research Ethics Committee of the Northern Savo Hospital District, Finland.

The researcher with qualitative research experience (ET) conducted the interviews using a semi-structured interview sheet. The interview included a broad range of open questions related to older people's lives during the COVID-19 pandemic. The interviews lasted 45–95 min each, and they were audiotape-recorded and transcribed verbatim. In the first phase, the data were analyzed using directed content analysis (23) seeking excerpts that were referring to personal social networks/social contacts. In the second phase, the codes were collated into five key categories representing different types of changes present in the data. The data analysis was carried out by the first and second author (JK, ET) first independently, and then discussing together the key categories and agreeing on the final findings. Excerpts representing each change type were selected and translated from Finnish to English.

## Results

The description of the interview participants is shown in Table 1. Participant characteristics are drawn from the COVID-19 survey (living situation, the current status of quarantine or physical isolation, and self-rated health,) and the CAIDE85+ main assessment [Mini-Mental-State Examination (MMSE) score (24)].

**Table 1:** Characteristics of the participants in the Cardiovascular Risk Factors, Aging, and Dementia (CAIDE) 85+^1^ COVID-19 qualitative in-depth interviews (*n* = 15).

**Age, mean (SD^2^)**	84.8 (7.3)
**Women, *n* (%)**	10 (66.7)
**Living alone, *n* (%)**	10 (66.7)
**Quarantine or physical isolation, *n* (%)** No quarantine or physical isolation Quarantine, self-imposed Avoiding physical contacts	5 (33.3) 0 (0) 10 (66.7)
**Self-rated health, *n* (%)** Poor Average or high	1 (6.7) 14 (93.3)
**Cognitive capacity, *n* (%)** MMSE^3^ score ≥ 25 MMSE^3^ score <25	13 (92.9) 1 (7.1)

1 *CAIDE85+ = Third follow-up of the Cardiovascular Risk Factors, Aging and Dementia study*.

2 *SD = Standard Deviation*.

3 *MMSE = Mini-Mental State Examination (24)*.

We were able to identify five types of changes in personal social networks of community-dwelling oldest old. On an individual level, one person could have had different types of changes during the pandemic. Table 2 summarizes these change types and their features in detail.

**Table 2:** Changes in personal networks among the community-dwelling oldest-old persons during the first and second wave of COVID-19 pandemic in Finland.

**Changes in personal networks during the first and second wave of Covid-19 pandemic among the oldest old persons**	**Features of each change type**
Type 1: Size of the personal network reduced significantly	•Avoiding all places with a lot of people •Fear (own or others) of the virus •Restricting contacts even with the closest family •Meetings outside impossible due to own of other person's sickness or disability •A relative/friend is a caregiver for someone else and cannot leave home •Hobbies has been closed •Use of digital tools were perceived as difficult and were not applied •Relatives prohibited contacting other people
Type 2: Personal networks remained the same, but modifications in contacting other people were done based on recommendations	•Phone contacts increased •Relatives, friends and neighbors were met outside and with safety distances •Video, internet and WhatsApp contacts with the family started •Applying safer ways of greeting and meeting people, i.e., not shaking hands anymore, using face masks •Hobbies, i.e., physical activity groups, organized online
Type 3: Personal networks increased during the pandemic	•Spending more time with partner •Contacting friends and relatives who had not been contacted for a long time •More frequent online contacts with children and grandchildren •Feeling socially more connected with the neighbors •Importance of pets increased
Type 4: Significant or unexpected change in personal network happened during the pandemic	•Death of a spouse •Death of a friend •Birth of great grandchildren
Type 5: The pandemic did not influence personal networks at all	•Phone contacts with relatives and friends were as common as previously •Friends and family visited regardless of restrictions or children live close or at the same house •Current personal social network was seen as fulfilling •Enjoying time alone and having no obligations to leave home

### Change Type 1: The Size of the Personal Network Reduced Significantly

We found that many interviewees had significantly reduced all their social contacts due to official restrictions laid down by the Finnish Government, and thus, the personal networks significantly diminished. Participants referred to the strong recommendations, which had instructed older adults aged 70 or over to stay at home and avoid all social contacts. Some interviewees did not meet even with the closest family members, as the following excerpts show, and sometimes their relatives had strongly asked them to stay at home or even prohibited contacting other people.

“*We have been quite isolated during this corona time” (male, 83 years)*

“*I didn't dare to meet even them [children] because of this corona. It's been a bit scary to meet people. I rather keep the two-meter distance to everyone” (female, 89 years)*

“*And there's of course been the age limit [70*+*] so you couldn't go anymore” (female, 93 years)*

“*We haven't really had any contacts outside of home. One of our children lives further and the other here [in the same city] but we didn't have any close contacts or meetings with them in the spring [2020]” (male, 81 years)*

“*Let's take it calmly but seriously at the same time. Follow the guidelines we have been given and avoid unnecessary contacts. Stay out of parties.” (male, 81 years)*

“*We have all agreed that we do not visit each other. We try to protect the small ones [newborns in the family] and us old people” (female, 87 years)*

The interviewees also reported that they started to avoid all public places with a lot of people. Based on the news from the media, they identified themselves as a high-risk group for serious COVID-19 infection which for some led to fear toward the virus. However, within our interviewees, most participants described friends and acquaintances as being more scared of the virus than themselves. This has led to a decrease in the interviewees' contacts too.

“*I have these friends who have shut themselves in their apartments. They don't let anyone in or they don't go anywhere. They have become more distant” (female, 91 years)*

“*If someone asks for someone to come and visit people say no, let's wait for things to calm down*. [...] *Before we had a lot of family and friends and we always visited them, but now, everyone is afraid to visit” (female, 82 years)*

Since the interviewees were aged 80 or over, physical illnesses or disabilities also acted as barriers to meeting relatives and friends outside of the home. One of the participants, an 89-year-old woman, described that she likes to go outside for walks but does not want to be a burden for others as she had to walk at a slow pace due to heart disease. Some interviewees reported having little or no contact because their friends or relatives were in poor health or taking care of others, such as a spouse and, therefore, unable to leave their homes during the pandemic.

“*She (friend) is kind of sick and cannot be outside, so she is at home a lot, or in other words only at home. She misses company a lot, so I have been calling her every now and then. And then these other friends of mine, pretty much all of them are six feet under or they have gone into such a bad shape that it is hard to be in contact even over the phone” (female, 89 years)*

“*Contact with my brother, well, he is so committed to be with his [sick] wife, previously we were in contact” (male, 88 years)*

Even though some of the participants' family members had suggested new ways of communication via digital platforms, phones, and the internet, learning new digital communication skills were perceived as difficult and, therefore, new tools were not applied. Some interviewees felt that they did not have enough cognitive capacity to use new technology or their attitude toward it was negative.

“*Well, we have not been using these video contacts. We tried but for some reason, it did not work, so I cannot use them” (male, 81 years)*

“*I also have this phone, the so-called smartphone. I have it even though I've said that I'm not that smart. It's not a good combination [laughing]. Yet, I think I could learn to use it but I'm not that enthusiastic to do so.” (female, 89 years)*

The interviewees also reported that their hobbies and people in these group activities had previously been an important part of personal network and social activity. The pandemic closed physical activity groups, meetings organized by different organizations, and other group activities, also in senior houses. The closing of these hobbies significantly reduced the possibility to meet other people. For some, such group activities had been the most important way to meet other people, and when these contacts were cut off, feelings of loneliness and loss increased.

“*We used to have a lot of activities in this house. A women's club got together once a week and then we had this balance training once a week. All sorts of things together*. [...] *We had a lot of these coffee parties together. Always some neighbors for coffee and things. But now we don't have them so much anymore and even all of these clubs and everything have ended completely” (female, 89 years)*

“*If you are active in several organizations you get social contacts from there. I like these kinds of contacts and now when they're completely missing it feels like a great loss and I feel lonely.” (male, 81 years)*

### Change Type 2: Personal Networks Remained the Same, but Modifications in Contacting Other People Were Done Based on Recommendations

Interviewees with this type of change appeared to cope well with the restrictions related to social contacts and applied multiple new ways of keeping in contact with relatives and friends. Especially phone contacts increased and almost all interviewees reported having regular phone contacts especially with close relatives, children, and grandchildren.

“*I talk with my daughter over the phone almost every day and then with these boys [her children] maybe a little less, but still often” (female, 89 years)*

“*These friends and relatives, they visited and called as often as before, but of course we needed to use telephone when it was not possible to meet” (female, 90 years)*

Distinct from the change type 1, interviewees with this change type reported that they had used videos, the internet, and WhatsApp to connect, especially with children and grandchildren. Many felt that these are easy ways to get in contact and they are still capable of learning new technology. Also, some hobbies, for example, physical activity groups, were organized online.

“*Now, we will have possibility to do physical activity when we got this big new television and from there, we will get exercise programs, morning workout and balance training, or that kind of training that you do while sitting on a chair” (female, 89 years)*

“*WhatsApp, WhatsApp is used by these younger ones and I have also learned how to use it.” (female, 89 years)*

“*I have received videos as well, video calls. I have had this computer, how should I say it” (female, 81 years)*

Interviewees with this change type reported that they did not completely stop seeing other people but tried to carefully follow the safety instruction by using face masks and avoiding shaking hands. In Finland, 2 m of safety distance when meeting other people was suggested, and the interviewees described how they tried to keep this in all social events. The meetings were held outside or at balconies instead of inside the houses or apartments. Due to the virus, many had found new ways of meeting and greeting each other so that contacts were still possible.

“*Yes, they [children] came. They had face masks on, but they came” (female, 91 years)*

“*Here in our neighborhood we know each other as we have lived here for so long. We met outside at the yard and kept a longer distance but could talk there for a half an hour even.” (female, 89 years age)*

“*And sometimes we did not care, we just hugged, And then when there was someone with whom we had not met for a while, we should have greeted each other with our elbows [laughing] but I said that let's rather touch each other on the shoulder or something”. (female, 89 years)*

“*Not shaking hands anymore, It's like a new custom you have learned and now it comes naturally”. (female, 81 years)*

“*We went to the sauna together, but in sauna, we were not sitting close to each other, it was like a bigger sauna where you could keep the two meters distance” (male, 83 years)*

“*Outside and with safety distances” (female, 91 years)*

“*We use the mask and hand sanitizer, we follow the recommendations and use these protective things where we go, that is how life is now” (female, 87 years)*

“*Well, at least, how do I say it, we keep distance to others and use masks and these protective matters (hand sanitizer), wash hands and everything” (female, 88 years)*

### Change Type 3: Personal Networks Increased During the Pandemic

The third change type included an increase in social contacts. Participants reported how the unexpected situation of the COVID-19 pandemic encouraged people to contact friends who they had not seen for a long time. In addition, some interviewees reported that their relationship with their spouse had become even more important and time spent with one's spouse had increased, which was seen as a positive change. Pets, especially dogs, were mentioned as one important source of social activity by many of the interviewees.

“*This corona time, it has kind of highlighted the contacts with grandchildren, they always call, I have no complaints” (female, 88 years)*

“*I have this one friend and we've been in contact less but when they said in the television that you have to remember old people I called her, even though she is a lot younger than me. I said that I remember you and I will surprise you and call”. (female, 81 years)*

“*Well, yes, over the phone, but the phone bills are higher [laughing], but of course over the phone we connected more and had long talks” (female, 89 years)*

“*I have of course my wife who takes so good care of me” (male, 83 years)*

“*I have more time with my spouse” (male, 81 years)*

“*I live alone with my dog, who is a big comfort and joy especially now during this corona time” (female, 90 years)*

### Change Type 4: Significant or Unexpected Change in Personal Network Happened During the Pandemic

Some of the participants reported that they had faced an unexpected loss of a spouse or a close friend during the pandemic and this naturally had a dominating effect on their personal network. They described how the death of a spouse had caused both psychological and physical symptoms which had affected everyday life, even though the death of an old person did not usually happen totally unexpectedly. On the other hand, new family members were born and the interviewees described how the birth of a new great-grandchild has brought joy to their lives and expanded their personal network.

“*My life situation changed in the summer very dramatically when my husband died. He left while sleeping by my side in early morning” (female, 89 years)*

“*A friend and close one who has left this life during this year, like my husband. Now just a month ago left one friend with whom we always used to call” (female, 90 years)*

“*This little girl, she has been a big joy for us during this autumn. She is five months old now and since she was ten days old, she has been visiting here at her great grandmother's place” (female, 87 years)*

### Change Type 5: The Pandemic Did Not Influence Personal Networks

The last type represents stability in personal networks. For some of the participants, the pandemic did not have a significant effect on personal networks. Many interviewees described how telephone contacts with relatives and friends remained as common as previously. Also, in many cases, closest relatives and friends visited older persons as often as previously, and it was considered relatively safe to meet in small groups.

“*This type of thing cannot revolutionize an old man's life” (male, 83 years)*

“*We have had [with the daughters] the same connection and contact as previously” (female, 90 years)*

“*Well, corona does not affect my life, at least now. Of course, if you visit the southern parts of Finland where people live more densely, like in Helsinki, where I have relatives. I always ask whether they have stayed healthy, and I have heard that anyone has not been affected” (female, 93 years)*

“*All my friends have called me like before and when they were allowed to visit again, they visited. I cannot say that anything has changed because my life has been really good anyway” (female, 90 years)*

Some interviewees told how they had never had a very large personal network and how they are now even happier with a smaller number of social contacts. Restrictions related to social connections were also seen in some cases as a relief because of not having any obligations to leave home.

“*I kind of enjoy being alone, I must say, and I have never cared so much about visitors” (female, 93 years)*

“*I am satisfied, I do not need anything more” (female, 91 years)*

## Discussion

This qualitative study with fifteen in-depth interviews among the oldest old community-dwelling persons living in Finland showed that older adults are heterogeneous in terms of their personal social networks and the effects of the global pandemic on their social relationships are diverse. We found that many interviewees took official recommendations seriously and tried to avoid all unnecessary social contacts. This, in most cases, led to a significant decrease in personal networks. This was commonly associated with fear of the virus. The results showed that all older people were not willing to use technology to compensate for the decrease in personal contacts. However, many interviewees felt that the personal networks remained relatively stable during the pandemic, but the forms of social contacts changed, for example, from physical meetings to phone and video contacts. Meetings also took place outside and only with a smaller amount of people at the same time.

In March 2020, the Finnish government recommended that all older people aged 70 and over should avoid all physical contact. Although many of our interviewees tried to follow this recommendation, other people were also met in person, mostly outside. It was also not uncommon that personal network in some forms increased during the pandemic, contacts with friends were activated and time spent with a spouse increased. For some interviewees, dramatic or unexpected change, for example, the death of a spouse or a friend was identified as a dominating factor that affected in many ways life during the pandemic. Finally, we also found that for some older adults, the global pandemic and its restrictions were not seen as a factor that modified their personal social networks. For some interviewees, life during the pandemic remained relatively stable and friends and family were visiting as often as previously. Especially for those who felt that they do not need a large personal network, the restrictions relating to the pandemic felt even relieving.

Existing research has widely shown that personal social networks are an important factor in well-being among older people but with increasing age, the quality of social contacts becomes more relevant than the quantity (25). A previous qualitative study by Vos et al. (26) investigated how older adults themselves experience changes in their social networks and how these changes impact their lives. This study was conducted before the pandemic, but interestingly similar changes as in the present study in the personal network were observed. For example, the sickness or death of a spouse was one of the most significant changes that caused sorrow and feelings of isolation. Diminishing personal network, whether caused by loss of significant people or by a change in own health or functioning was considered as a crisis that was described to turn life upside-down (26). In the present study, we found that during the pandemic, the loss of a spouse or a friend or declining health led to changes in their personal network, and these changes can be considered not to be pandemic-related. However, COVID-19 resulted in numerous new obstacles for maintaining social connections that to our knowledge have not been reported previously, for example, fear of compromising own or others' health, requirements to use technology, or meet outside where safety distances can be maintained.

Some of the interviewees described how diminished personal network had increased feelings of loneliness. Also, other studies have found that older adults who have been socially isolated have especially reported increased rates of loneliness during the pandemic. A large population-based study from the US reported that one-third of the population aged 55 and older experienced feelings of loneliness during Spring 2020. Especially persons who were not married or in a relationship commonly reported loneliness (27). Similar results have been reported from Europe in the study by Cohn-Schwartz et al. (28), who found that especially older people who followed the recommendations and were physically isolated felt lonelier during the pandemic than before (28). However, larger social networks, a higher number of social interactions both before and during the pandemic, living with someone, and perceived availability of social support are protective against loneliness (16). Among older people, ways to cope with feelings of loneliness are numerous, such as increasing physical engagement, using technologies, and also acceptance, positive attitude, religion, and spirituality (29). Our results also showed that while physical engagement with other people was not possible all the time, connections especially with family members were compensated using technology and many interviewees seemed to cope well and accept the existing situation without a noticeable negative attitude.

Toward the use of digital technology to compensate for the decline in a personal network, the interviewees had differing perspectives and opinions. Although our study population consisted of people aged 80 and over, we found that adopting new technology in order to maintain personal networks during the time of restrictions was relatively common, and the attitudes toward using digital tools were positive. Other studies are in line with our results by showing that during the pandemic, especially oldest old women and persons with high cognitive abilities have been able to actively use the internet (30). The studies conducted before the pandemic have shown that frequent social media contacts are associated with lower levels of loneliness and perceived social support (31). Similarly, during these exceptional times of social isolation, internet use aiming to maintain communications with other people, such as video calls, increases the perceived quality of life among older people (32). Furthermore, Rolandi et al. (33) found that among community-dwelling oldest-old individuals in Lombardy, Italy, those persons who had received training for using social networking sites, had less reduction in their social contacts due to the COVID-19 pandemic as compared with individuals who had not received training (33). However, it should be noted that in developed countries, replacing social contacts by using the internet and other digital tools is much easier than in developing countries. This was shown in a study by Ekoh et al. (34), which found that during the COVID-19 pandemic older people in rural Nigeria were digitally excluded (34). Since the oldest old individuals are a very heterogeneous group in terms of technology use, individualized guidance for those who are willing to learn new communication ways should be provided, but also it should be noted that the use of technology does not suit all and many of the oldest old persons may be easily left out if there is an assumption that all communication can be maintained using technology. There is currently a lack of strong evidence of digital technology interventions on reducing loneliness in older people (35) and, therefore, supporting safe ways of physical contact remains important.

The importance of having connections with neighbors was mentioned by many of our interviewees and many older persons also received help from the neighbors. Similar findings were reported by Brown et al. (36), who found that during the COVID-19 lockdown over half of community-dwelling older people aged 76–97 years increased engagement with their neighbors, and own garden was mentioned as one enjoyable aspect of the lockdown, providing possibilities to chat with neighbors (36). It has also been previously reported that for older people, neighbors are often an important source of information related to the COVID-19 situation (37).

Having social contacts and being socially active are known to protect from several adverse health events. However, it should be remembered that like younger people, also oldest old people are heterogeneous in terms of their preferences and wishes for personal networks and social contacts. For some people, a socially active lifestyle feels tiring and extensive social contact may even have a negative effect on their perceived well-being. It has been hypothesized that extroverts who are used to having an active social life would suffer more from changes related to personal networks during the pandemic and it is possible that personality modifies the feelings related to the pandemic and also a degree of suffering from isolation. A study by Gubler et al. (38) found that while extraversion usually protects from loneliness, extraversion seems to be associated with feelings of loneliness in the time of pandemic-related restrictions and the authors suggested that extraversion may lose some of its protective value for loneliness and well-being when opportunities to engage in social activities are limited (38). Also, a large multi-national study which was based on survey data from over 90,000 respondents suggested that the lifestyle associated with restrictions may feel more natural to introverts than to extroverts (39). Also, for some of our interviewees, instead of having contact with other people, pets in our data especially dogs, seemed to have a special role in their personal social networks. Similar findings were found in the study by Carr et al. (40) who suggested that there may be even a potential therapeutic effect of dog walking for the promotion of mental health in older people and the effect may have been more pronounced during the COVID-19 pandemic (40).

Our results on the heterogeneity of changes in social relationships during the pandemic are in line with the earlier findings from our research group. Among the Finnish older population, 55% reported a decrease in contacts with friends and relatives during the first wave of the pandemic, for 34% the contacts remained the same, and 11% reported an increase in contacts (41). When looking at older persons' activity in general, Rantanen et al. (43) have reported that among older Finnish adults, active aging score (42), which describes broadly everything that people do, was significantly lower during the first wave of the pandemic as compared to 2 years before (43).

This study has some limitations. First, it should be noted that the COVID-19 pandemic has shown to have different psychological responses based on culture (44), and also based on the specific restriction measures as explained earlier. Our study population consisted of the oldest old community-dwelling people from a relatively limited area in Eastern Finland and therefore these results cannot be generalized to older people in general or to older people from other countries. In the area where the data was collected, the incidence rate for COVID-19 was low during the data collection. This may be reflected in the way the interviewees describe the changes in their personal networks but most likely not in the types of changes *per se*. Second, the interview participants represent a fairly homogenous group based on their background factors, such as socioeconomic status, ethnicity, and living environment. But still, as the results show, the impacts of the COVID-19 pandemic have been highly diverse even in this group. The strengths of this study also include shedding light on the experiences of the oldest old, which are often left out of research and public discussion.

Along with the present study, emerging evidence has shown that the pandemic influences the oldest old individuals in numerous ways. The current situation has increased the risk for decline in physical and mental health (16, 30) and also challenged the possibilities to engage with other people. Restrictions and isolation are associated with adverse health outcomes, including increased risk for dementia (45) are, therefore, possibilities to maintain a socially active lifestyle should be supported among the oldest old people. However, many older adults have coped well during these exceptional times and have been able to adopt new ways of communication or live their lives just like before the pandemic. Due to the large individual variety, when planning policy actions and interventions to support social participation and activity, this heterogeneity of older people should be taken into account. When aiming to improve the health and well-being of oldest-old persons, one size does not fit all. It is important to provide support and encouragement to social activity and help to maintain personal networks, but first, it is essential to identify the individual needs of the oldest old.

## Data Availability Statement

The datasets presented in this article are not readily available because access to the data is maintained by the CAIDE research team. Requests to access these datasets should be directed to Mariagnese Barbera, mariagnese.barbera@uef.fi.

## Ethics Statement

The studies involving human participants were reviewed and approved by the Research Ethics Committee of the Northern Savo Hospital District. The patients/participants provided their written informed consent to participate in this study.

## Author Contributions

JK: conceptualization, methodology, data analyses, resources, writing—original draft, writing—review and editing, funding acquisition, and project administration. ET: conceptualization, methodology, data analyses, data curation, writing—original draft, and writing—review and editing. IL: conceptualization, writing—original draft, writing—review and editing, and funding acquisition. TN: resources, data curation, project administration, and writing—review and editing. MK: resources, project administration, funding acquisition, and writing—review and editing. AS: resources, data curation, project administration, funding acquisition, and writing—review and editing. All the authors contributed to the article and approved the submitted version.

## Funding

This study was financially supported by the Juho Vainio Foundation (Finland), Finnish Cultural Foundation, Yrjö Jahnsson Foundation (Finland), Ministry of Culture and Education (Finland), and Academy of Finland (Grant Numbers 335524, 317465, 287490, and 334419).

## Conflict of Interest

The authors declare that the research was conducted in the absence of any commercial or financial relationships that could be construed as a potential conflict of interest.

## Publisher's Note

All claims expressed in this article are solely those of the authors and do not necessarily represent those of their affiliated organizations, or those of the publisher, the editors and the reviewers. Any product that may be evaluated in this article, or claim that may be made by its manufacturer, is not guaranteed or endorsed by the publisher.
